# Macrophage inducible nitric oxide synthase promotes the initiation of lung squamous cell carcinoma by maintaining circulated inflammation

**DOI:** 10.1038/s41419-018-0653-3

**Published:** 2018-05-29

**Authors:** Zane Gray, Gongping Shi, Xin Wang, Yinling Hu

**Affiliations:** 1 Cancer and Inflammation Program, Center for Cancer Research, National Cancer Institute, National Institutes of Health, Frederick, Maryland 21701 USA; 2 Department of Cell & Molecular Biology, Tulane University, 2000 Percival Sterm Hall, 6400 Freret Street, New Orleans, LA 70118 USA; 3 The Respiratory Department, Jinan Central Hospital Affiliated to Shandong University, Jinan, Shandong 250013 People’s Republic of China

The role of macrophage-inducible nitric oxide synthase (NOS2 or iNOS) in carcinogenesis is controversial, although epithelial cell NOS2 has been shown to promote carcinogenesis^1,2^. IL-1, TNFα, IFNγ, and lipopolysaccharide (LPS) all induce NOS2 expression in macrophages, but IL-4 and IL-13, which are both M2 cytokines, repress NOS2 expression in macrophages. NOS catalyzes l-arginine to produce nitric oxide (NO) and l-citrulline. NOS2 expression in macrophages is minimal but is locally induced to high-output quantities of NO at a micromolar range for prolonged periods of time^3^. Xiao et al.^4^ reported elevated NOS2 expression in pulmonary infiltrating macrophages in kinase-dead *Ikkα* knock-in (*L-Ikkα*^*KA/KA*^, *KA/KA*) mice that develop spontaneous lung squamous cell carcinoma (SCC) driven by IKKα reduction and increased infiltrating macrophages overexpressing NOS2. Our unpublished data showed elevated expression levels of NOS2 in the monocytes of lung SCC patients compared to non-cancer people. Recently, Wang et al.^5^ demonstrated that NOS2 ablation or NOS2 null bone marrow (BM) transfer significantly reduces DNA damage, inflammation, and lung SCC incidence, which shows that macrophage NOS2 induction is not only a response to an inflammatory microenvironment but also a promoter of lung carcinogenesis (Fig. 1a).

**Fig. 1:**
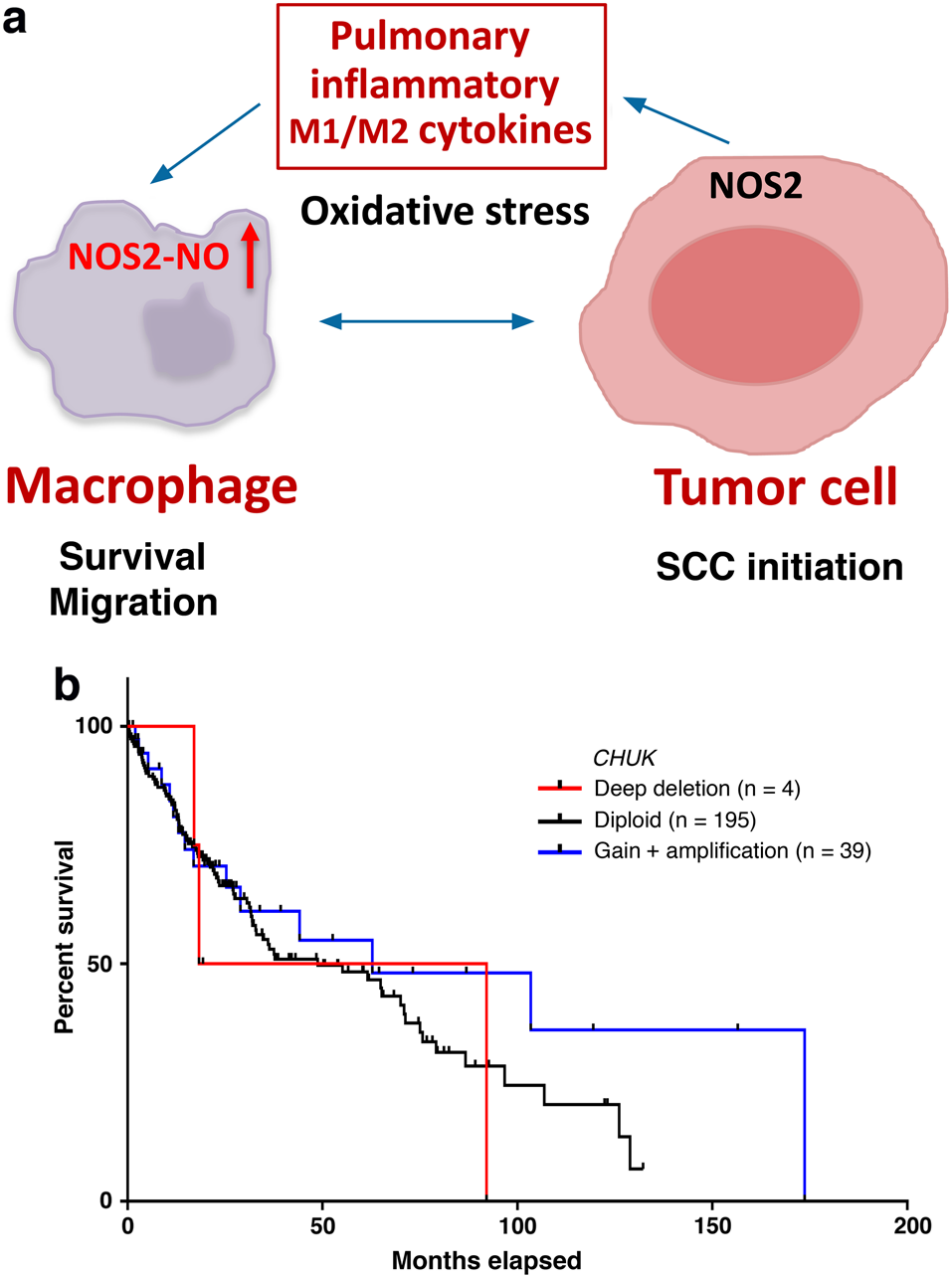
**a** A model showing how increased macrophage NOS promotes lung squamous cell carcinoma (SCC) development, enhances macrophage survival and migration, increases oxidative stress, and circulates or maintains inflammatory signals between epithelial cells and macrophages. **b** Survival curves for patients with lung SCCs bearing *CHUK* alterations, including deep deletion (*n* = 4), diploid (*n* = 195), and gain and amplification (*n* = 39) (cBioPortal for Cancer Genomics). *n* patient numbers. *p* = 0.227, *χ*^2^ test

NO is essential for many cellular events, but excessive NO damages cells and organs and interacts with the intermediate components of reactive oxygen species (ROS), which further elevate oxidative stresses and provoke inflammation. Thus, the level of NOS2 may reflect the status of local inflammation. Arginase-1, an M2 macrophage marker, catalyzes L-arginine to produce L-ornithine and urea so that arginase-1 and NOS2 compete for l-arginine. Because NOS2 depletion significantly decreases lung SCC incidence in *KA/KA;Nos2*^−/−^ mice^5^, this model offers an opportunity to elucidate the mechanism underlying macrophage NOS2’s function in lung carcinogenesis. Wang et al.^5^ further showed that WT, *KA/KA*, and *KA/KA;Nos2*^−/−^ macrophages in vitro do not express NOS2 and that LPS treatment induces a comparable level of NOS2 in WT and *KA/KA* macrophages but not in *KA/KA;Nos2*^−/−^ macrophages (a negative control). Therefore, increased NOS2 induction in the macrophages of *KA/KA* lungs is likely due to increased inflammatory cytokines in *KA/KA* lungs. NOS2 ablation decreases infiltrating macrophage numbers, pulmonary inflammation, and lung SCC incidence^5^, suggesting that macrophage NOS2 maintains an elevated inflammatory status that promotes carcinogenesis.

Intriguingly, *KA/KA* and *KA/KA;Nos2*^−/−^ macrophages unexpectedly express a comparable level of arginase-1, suggesting that reduced SCC incidence is not correlated with decreased macrophage arginase-1 levels^5^. Although *KA/KA* macrophages express increased levels of many cytokines compared to *KA/KA;Nos2*^−/−^ macrophages, the expression level of IL-13 and IL-4 is higher in *KA/KA;Nos2*^−/−^ than in *KA/KA* macrophages, suggesting that macrophage NOS2’s effect on tumor promotion is not through altering M1 and M2 macrophage features^5^. Instead, increasing macrophage infiltration, macrophage survival and migration, and expression of multiple cytokines and chemokines is positively associated with lung SCC development. Furthermore, Wang et al.^5^ identified foamy macrophages characterized with impaired lipid metabolism that result in cytoplasmic needle-shaped crystalline bodies, greatly enlarged cell sizes, and elevated Ym-1 levels (or chitinase-3-protein 3, Chi3l3)^6^, another M2 marker, in *KA/KA* lungs. NOS2 deletion reduces the foamy macrophage and total macrophage number associated with decreased lung SCC incidence. It has been reported that cigarette smoke can lead to lipid accumulation in macrophages^7^. We still do not know whether foamy macrophages are present in human lung SCCs, if lung SCCs promote foamy macrophage formation or if foamy macrophages facilitate lung SCC development, and whether IKKα inactivation promotes foamy macrophage formation.

Lung SCC and adenocarcinoma (ADC) are two major types of human lung cancer. We have shown that deletions of the *CHUK* locus that encodes IKKα significantly reduce the survival time of human patients with *KRAS* mutation lung ADCs as well as total lung ADCs^8^. *CHUK* deletions are indeed found in human lung SCCs and show a tendency toward the reduced survival time, whereas patients with lung SCCs expressing increased IKKα show prolonged survival time (cBioPortal for Cancer Genomics; Fig. 1b). Consistently, IKKα reduction promotes but elevated IKKα expression in keratinocytes inhibits chemical carcinogen-induced skin SCC development in mice^9–11^. IKKα reduction and increased pulmonary inflammation drive the development of lung SCCs characterized with the hallmarks of human lung SCC, including keratin 5 (K5), Ki67, p63, and TRIM29 in *KA/KA* mice. The *KA/KA* SCCs express downregulated p53, Rb, and LKB1, elevated p-EGFR, p-ERK, CDK1, and DNA damage; and marked pulmonary macrophage infiltration, all of which are frequently detected in human lung SCCs^5^. Therefore, studying IKKα-associated lung SCC development is of medical significance. Cigarette smoke, an etiological cause of human lung SCC, induces DNA damage, inflammation that recruits macrophages, and NOS2 expression. *KA/KA* mice develop autoinflammation^12^ so that marked macrophage infiltration and enhanced cytokine and chemokine expression levels are present in the lungs of *KA/KA* mice at four weeks of age, prior to the SCC formation^4^. Therefore, lung SCC development is driven by increased macrophages/inflammation and IKKα reduction, while NOS2 induction contributes to the pathogenic activity of *KA/KA* macrophages.

To determine the effect of lung epithelial cell NOS2 or macrophage NOS2 on lung SCC development, Wang et al.^5^ performed BM transplantations by injecting *KA/KA* BM or *KA/KA;Nos2*^−^^/−^ BM into irradiated *KA/KA;Nos2*^−/−^ mice or *KA/KA* mice. All *KA/KA* mice receiving *KA/KA* BM developed lung SCCs (positive controls), while all *KA/KA;Nos2*^−/^^−^ mice receiving *KA/KA;Nos2*^−/^^−^ BM did not develop tumors (negative controls). Lung SCC incidence is significantly decreased in chimeric *KA/KA* mice receiving *KA/KA;Nos2*^−/^^−^ BM as well as in chimeric *KA/KA;Nos2*^−/^^−^ mice receiving *KA/KA* BM, demonstrating that both macrophage NOS2 and epithelial cell NOS2 are required for carcinogenesis. In conclusion, Wang et al.^5^ reported that macrophage NOS2 promotes lung SCC initiation by maintaining circulated inflammatory responses between macrophages and lung epithelial cells, while macrophage NOS2 deletion decreases lung SCC incidence.

## References

[1] Okayama H (2013). Int. J. Cancer.

[2] Liu CY (1998). Br. J. Cancer.

[3] Mattila JT (2014). Front. Immunol..

[4] Xiao Z (2013). Cancer Cell..

[5] Wang, X. et al. *Cell Death Dis*. 10.1038/s41420-018-0046-5(2018).

[6] Roszer T (2015). Mediat. Inflamm..

[7] Morissette MC (2015). Eur. Respir. J..

[8] Song NY (2018). Proc. Natl Acad. Sci. USA.

[9] Liu B (2006). Proc. Natl Acad. Sci. USA.

[10] Park E (2007). Cancer Res..

[11] Xia X (2010). Am. J. Pathol..

[12] Zhu F (2017). Cell. Host. Microbe.

